# Surveillance for Acute Flaccid Myelitis ― United States, 2018–2022

**DOI:** 10.15585/mmwr.mm7304a1

**Published:** 2024-02-01

**Authors:** Erin R. Whitehouse, Adriana Lopez, Randall English, Halle Getachew, Terry Fei Fan Ng, Brian Emery, Shannon Rogers, Sarah Kidd

**Affiliations:** ^1^Division of Viral Diseases, National Center for Immunization and Respiratory Diseases, CDC; ^2^Tanaq Management Services, Anchorage, Alaska.

**Summary**

**What is already known about this topic?**

Acute flaccid myelitis (AFM) is a serious neurologic condition that has been associated with enterovirus-D68 (EV-D68) infection. Biannual increases in cases were observed in the United States during 2014, 2016, and 2018.

**What is added by this report?**

The number of AFM cases has remained low since 2018, including during 2022, when an increase in EV-D68 respiratory disease was observed.

**What are the implications for public health practice?**

Why increased EV-D68 circulation in 2022 was not associated with an increase in AFM cases or when AFM will peak again is unknown. Clinicians should remain alert for cases of AFM to provide timely clinical care, report cases to public health departments, and collect appropriate specimens.

## Abstract

Acute flaccid myelitis (AFM) is a serious neurologic condition primarily affecting children; AFM can cause acute respiratory failure and permanent paralysis. AFM is a rare but known complication of various viral infections, particularly those of enteroviruses (EVs). Increases in AFM cases during 2014, 2016, and 2018 were associated with EV-D68 infection. This report examines trends in confirmed AFM cases during 2018–2022 and patients’ clinical and laboratory characteristics. The number of AFM cases was low during 2019–2022 (28–47 cases per year); the number of cases remained low in 2022 despite evidence of increased EV-D68 circulation in the United States. Compared with cases during the most recent peak year (2018), fewer cases during 2019–2021 had upper limb involvement, prodromal respiratory or febrile illness, or cerebrospinal fluid pleocytosis, and more were associated with lower limb involvement. It is unclear why EV-D68 circulation in 2022 was not associated with an increase in AFM cases or when the next increase in AFM cases will occur. Nonetheless, clinicians should continue to suspect AFM in any child with acute flaccid limb weakness, especially those with a recent respiratory or febrile illness.

## Introduction

Acute flaccid myelitis (AFM) is a serious neurologic condition that causes paralysis often requiring intensive care and mechanical ventilation and can lead to severe sequelae and disability. Many pathogens can cause AFM. Laboratory and surveillance data suggest that enteroviruses (EVs), particularly EV-D68, are a common cause; EV-D68 was associated with peaks in U.S. AFM cases during 2014, 2016, and 2018 (*1*). Since 2014, CDC has conducted surveillance for AFM, including laboratory testing and typing of EV-positive samples to better understand the demographic and clinical characteristics and possible causes of AFM. This report updates AFM surveillance data since 2018, the most recent reported peak year for AFM.

## Methods

As part of national surveillance for AFM, U.S. health departments report cases of acute flaccid limb weakness with any spinal cord gray matter lesion on magnetic resonance imaging to CDC. Health departments complete and submit a patient summary form, which includes demographic and clinical information and important elements from the patient’s medical record. In addition, health departments and clinicians submit available cerebrospinal fluid (CSF), respiratory, serum, and stool specimens for laboratory testing. At CDC, specimens are tested for EV/rhinovirus (EV/RV) using real-time polymerase chain reaction*; EV/RV–positive specimens are molecularly typed using protocols that have been previously described^†^ (*2*,*3*). For surveillance purposes, confirmed AFM is defined as acute flaccid limb weakness accompanied by magnetic resonance imaging demonstrating a spinal cord lesion largely restricted to gray matter and spanning one or more vertebral segments (*4*).

Case reports have been used to describe trends in confirmed AFM cases since surveillance began in August 2014. For this study, patient summary forms, medical records, and laboratory data were analyzed to describe patient and case characteristics in 2018, the most recent peak year, through 2022. Reported EV/RV data include laboratory results that were documented in records sent to CDC as well as results of testing performed at CDC. This activity was reviewed by CDC, deemed not research, and was conducted consistent with applicable federal law and CDC policy.^§^

## Results

### Characteristics of Patients with Confirmed AFM

During 2018, 2019, 2020, 2021, and 2022, a total of 238, 47, 33, 28, and 47 confirmed AFM cases, respectively, were reported to CDC (Figure) (Table 1). The proportion of patients with confirmed AFM aged <18 years decreased from 94% in 2018 to 81% in 2022. Among patients aged <18 years, the median age was lower in 2018 (5.1 years) than that during 2019 (6.3 years), 2020 (8.0), 2021 (8.0), and 2022 (7.1). During 2018, 92% of patients with confirmed AFM experienced a prodromal respiratory or febrile illness, 84% had upper limb involvement compared with 56% with lower limb involvement, and 87% had CSF pleocytosis. These features were still common among patients with confirmed AFM during 2019–2022; however, compared with 2018, a lower proportion of patients with confirmed AFM during 2019–2021 had a prodromal respiratory or febrile illness (57%–64%), upper limb involvement (58%–74%), or CSF pleocytosis (42%–49%). In 2022, the proportion of patients with a prodromal respiratory or febrile illness (79%), upper limb involvement (74%), and CSF pleocytosis (68%) was lower than that in 2018 but higher than the proportions during 2019–2021. In addition, during 2019–2021, a higher proportion of patients had lower limb involvement (74%–93%) than patients during 2018 (56%) and 2022 (64%) did.

**FIGURE:**
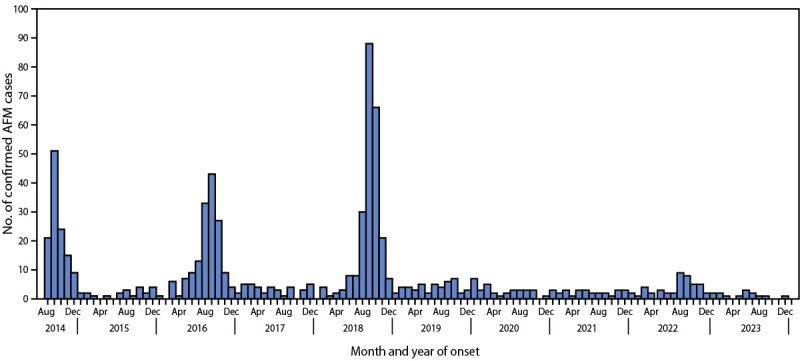
Confirmed cases of acute flaccid myelitis, by month and year of onset (N = 741) — United States, August 2014–January 2024* **Abbreviation:** AFM = acute flaccid myelitis. *As of January 26, 2024.

**TABLE 1:** Demographic and clinical characteristics of patients with confirmed acute flaccid myelitis ― United States, 2018–2022*

Characteristic	Year, no. (%)				
	2018 N = 238	2019 N = 47	2020 N = 33	2021 N = 28	2022 N = 47
**Age group, yrs**					
<18	223 (94)	43 (92)	30 (91)	23 (82)	38 (81)
Median age, all patients, yrs (IQR)	5.3 (3.3–8.2)	6.6 (2.9–12.8)	9.2 (3.1–14.5)	9.3 (5.2–16.1)	11.0 (5.8–14.3)
Median age <18, yrs (IQR)	5.1 (3.2–7.6)	6.3 (1.6–11.4)	8.0 (2.8–11.9)	8.0 (4.6–13.2)	7.1 (5.1–11.7)
**Sex**					
Female	100 (42)	32 (68)	16 (48)	16 (57)	21 (45)
Male	138 (58)	15 (32)	17 (52)	12 (43)	26 (55)
**Race and ethnicity** ^†^					
AI/AN	0 (—)	0 (—)	0 (—)	0 (—)	0 (—)
Asian	8 (3)	2 (4)	3 (9)	0 (—)	3 (6)
Black or African American	21 (9)	7 (15)	4 (12)	2 (7)	5 (11)
NH/OPI	1 (—)	0 (—)	0 (—)	0 (—)	0 (—)
White	125 (53)	18 (38)	12 (36)	11 (39)	27 (57)
Hispanic or Latino	47 (20)	12 (26)	9 (27)	10 (36)	6 (13)
Multiracial	4 (2)	1 (2)	0 (—)	1 (4)	1 (2)
Unknown	32 (13)	7 (15)	5 (15)	4 (14)	5 (11)
**U.S. Census Bureau region^§^**					
Northeast	41 (17)	3 (6)	6 (18)	8 (29)	10 (21)
Midwest	61 (26)	7 (15)	7 (21)	4 (14)	8 (17)
South	80 (34)	18 (38)	12 (36)	10 (36)	13 (28)
West	56 (24)	19 (40)	8 (24)	6 (21)	16 (34)
**Limbs affected**					
Upper	200 (84)	35 (74)	19 (58)	18 (64)	35 (74)
Lower	133 (56)	35 (74)	27 (82)	26 (93)	30 (64)
**Illness during the 4 weeks before onset of limb weakness**					
Any illness	223 (94)	32 (68)	21 (64)	20 (71)	39 (83)
Any respiratory illness	187 (79)	23 (49)	15 (45)	13 (46)	29 (62)
Any fever	174 (73)	14 (30)	13 (39)	8 (29)	22 (47)
Any respiratory illness or fever	218 (92)	27 (57)	21 (64)	16 (57)	37 (79)
Any gastrointestinal illness	80 (34)	12 (26)	3 (9)	9 (32)	12 (26)
**Timing of preceding illness, median days before limb weakness**^¶^ **(IQR)**					
Any illness	5 (3–8)	4 (3–7.5)	5.5 (2–11.5)	6 (2.5–11)	5.5 (2.8–8.2)
Any respiratory illness	5 (3–8)	5 (3–14)	6 (2.5–13.5)	4.5 (3–8)	6 (4–8)
Any fever	3 (1–5)	3 (2–5.8)	2 (1–5.5)	2.5 (1.8–15)	3 (1–8.2)
Any respiratory illness or fever	5 (3–7)	4 (2.5–7)	5.5 (2–11.5)	4.5 (2.8–9.8)	5.5 (3–8)
Any gastrointestinal illness	2 (1–6)	3.5 (2–8)	4 (0–14)	2 (0–7)	1.5 (0–9.2)
**CSF microscopic examination, no./No. (%)**					
CSF pleocytosis	183/210 (87)	21/43 (49)	13/27 (48)	11/26 (42)	28/41 (68)
Median WBC count, cells/mm^3^ (IQR)**	95 (43–163)	107 (41.5–209.5)	36 (9–72)	38 (13–94)	56.5 (26.2–77)
**Hospitalization and clinical care**					
Hospitalized	233 (98)	46 (98)	33 (100)	28 (100)	46 (98)
**Timing of hospitalization relative to onset of limb weakness, no./No. (%)**					
Before	24/233 (10)	6/46 (13)	1/33 (3)	0/28 (—)	3/46 (7)
After	208/233 (89)	40/46 (87)	32/33 (97)	28/28 (100)	43/46 (93)
Unknown	1/233 (—)	0/46 (—)	0/33 (—)	0/28 (—)	0/46 (—)
**Days from onset of weakness to hospitalization (among those hospitalized after onset of weakness), no./No. (%)**					
Median (IQR)	1 (0–2)	1 (0–1)	1 (1–1)	1 (0–1)	1 (1–3)
0–1	135/208 (65)	33/40 (82)	27/32 (84)	23/28 (82)	26/43 (60)
2–3	52/208 (25)	5/40 (12)	4/32 (12)	3/28 (11)	11/43 (26)
4–7	10/208 (5)	2/40 (5)	0/32 (—)	1/28 (4)	4/43 (9)
>7	11/208 (5)	0/40 (—)	1/32 (3)	1/28 (4)	2/43 (5)
**Treatment**					
Steroids, no IVIG	55 (23)	14 (30)	8 (24)	2 (7)	11 (23)
IVIG, no steroids	54 (23)	12 (26)	6 (18)	8 (29)	10 (21)
Both steroids and IVIG	81 (34)	15 (32)	14 (42)	17 (61)	25 (53)
Plasma exchange	32 (13)	10 (21)	11 (33)	7 (25)	12 (26)
Admitted to ICU	129 (54)	24 (51)	20 (61)	21 (75)	24 (51)
Respiratory support	65 (27)	16 (34)	6 (18)	8 (29)	11 (23)
Mechanical ventilation	55 (23)	13 (28)	5 (15)	7 (25)	9 (19)
**Location of first medical encounter after onset of weakness**					
Emergency department	134 (56)	32 (68)	24 (73)	19 (68)	30 (64)
Primary care provider	49 (21)	4 (9)	4 (12)	2 (7)	4 (9)
Urgent care provider	16 (7)	4 (9)	1 (3)	3 (11)	2 (4)
Weakness onset during inpatient hospitalization	24 (10)	6 (13)	1 (3)	0 (—)	3 (6)
Unknown or other	15 (6)	1 (2)	3 (9)	4 (14)	8 (17)
**Days from onset of weakness to first medical encounter (excluding those hospitalized before onset of weakness), no./No. (%)**					
Median (IQR)	0 (0–1)	0 (0–1)	0 (0–0)	0 (0–1)	0 (0–1)
0–1	161/213 (76)	36/41 (88)	31/32 (97)	25/28 (89)	33/44 (75)
2–3	33/213 (15)	3/41 (7)	0/32 (—)	2/28 (7)	6/44 (14)
4–7	4/213 (2)	0/41 (—)	0/32 (—)	1/28 (4)	2/44 (5)
>7	3/213 (1)	2/41 (5)	0/32 (—)	0/28 (—)	0/44 (—)
Unknown	12/213 (6)	0/41 (—)	1/32 (3)	0/28 (—)	3/44 (7)

**Abbreviations:** AI/AN = American Indian or Alaska Native; CSF = cerebrospinal fluid; ICU = intensive care unit; IVIG = intravenous immunoglobulin; NH/OPI = Native Hawaiian or other Pacific Islander; WBC = white blood cell.

* Table includes updated demographic and clinical information and supersedes previously published data. https://doi.org/10.15585/mmwr.mm7044a2

^†^ Persons of Hispanic or Latino (Hispanic) origin might be of any race but are categorized as Hispanic; all racial groups are non-Hispanic.

^§^ https://www2.census.gov/geo/pdfs/maps-data/maps/reference/us_regdiv.pdf

^¶^ Timing calculated among cases with the prodromal illness or symptom and documented valid dates of onset.

** Median cells/mm^3^ was calculated among cases with CSF pleocytosis (>5 WBC/mm^3^).

During all years, nearly all (98%–100%) patients with confirmed AFM were hospitalized (Table 1). The majority were hospitalized within 1 day of onset of weakness, and an emergency department was the most common location of the first medical encounter after the onset of weakness (56%–73% of patients). More than one half of patients (51%–75%) were admitted to an intensive care unit during hospitalization, 18%–34% of all patients required some form of respiratory support, and 15%–28% of all patients received mechanical ventilation.

### Detection of EV/RV in Patients with Confirmed AFM

EV/RVs were detected in specimens from at least one anatomic site in 50% of patients who were tested for EV/RV in 2018, 39% in 2019, 28% in 2020, 43% in 2021, and 50% in 2022 (Table 2). In 2018, the most common EV detected among patients with confirmed AFM was EV-D68 (37), with the majority of detections identified from respiratory specimens. In contrast, EV-D68 was detected in one patient in 2019 and no patients during 2020–2022. In addition, EV-A71 was detected among 13 patients in 2018, two in 2019, one each in 2020 and 2021, and two in 2022.

**TABLE 2:** Enterovirus/rhinovirus results from respiratory, stool, cerebrospinal fluid, and serum specimens collected from patients with confirmed acute flaccid myelitis — United States, 2018–2022*

Specimen source	Year, no. (%)				
	2018 N = 238	2019 N = 47	2020 N = 33	2021 N = 28	2022 N = 47
**Any source^†^**					
All patients with results	224 (94)	44 (94)	32 (97)	28 (100)	44 (94)
Patients with positive results	112 (50)	17 (39)	9 (28)	12 (43)	22 (50)
**EV/RV type results^§^**					
EV-D68	37	1	0	0	0
EV-A71	13	2	1	1	2
RVs	10	1	3	3	3
Other typed EVs^¶^	8	2	0	2	2
Unknown or not typed	46	11	5	6	15
**Respiratory^†^**					
All patients with results	195 (82)	40 (85)	29 (88)	27 (96)	37 (79)
Patients with positive results	97 (50)	14 (35)	8 (28)	9 (33)	18 (49)
**EV/RV type results^§^**					
EV-D68	37	1	0	0	0
EV-A71	11	0	0	0	0
RVs	10	1	3	3	3
Other typed EVs^¶^	1	0	0	0	0
Unknown or not typed	40	12	5	6	15
**Stool**					
All patients with results	112 (47)	24 (51)	15 (45)	14 (50)	26 (55)
Patients with positive results	25 (22)	7 (29)	2 (13)	3 (21)	6 (23)
**EV/RV type results^§^**					
EV-D68	3	0	0	0	0
EV-A71	2	2	1	1	2
RVs	0	0	0	0	0
Other typed EVs^¶^	7	2	0	2	2
Unknown or not typed	13	3	1	0	2
**Cerebrospinal fluid**					
All patients with results	191 (80)	40 (85)	30 (91)	27 (96)	37 (79)
Patients with positive results	9 (5)	0 (—)	0 (—)	1 (4)	1 (3)
**EV/RV type results^§^**					
EV-D68	2	0	0	0	0
EV-A71	1	0	0	0	0
RVs	0	0	0	0	0
Other typed EVs	0	0	0	0	0
Unknown or not typed	6	0	0	1	1
**Serum**					
All patients with results	109 (46)	30 (64)	23 (70)	21(75)	24 (51)
Patients with positive results	4 (4)	0 (—)	1 (4)	2 (10)	0 (—)
**EV/RV type results^§^**					
EV-D68	1	0	0	0	0
EV-A71	0	0	1	0	0
RVs	0	0	0	0	0
Other typed EVs^¶^	2	0	0	1	0
Unknown or not typed	1	0	0	1	0

**Abbreviations:** EV = enterovirus; RV = rhinovirus.

* Table includes updated laboratory information and supersedes previously published data. https://doi.org/10.15585/mmwr.mm7044a2

^†^ Some patients had multiple positive specimens. In addition, respiratory coinfection with two EV/RV types was detected in two cases in 2018 (EV-D68 and Echovirus 6 in one case and EV-D68 and RV-A2 in the other case).

^§^ Percentage not calculated.

^¶^ Other EVs identified in confirmed AFM patients included Echovirus 11 (one patient each in 2018, 2019, and 2021), Echovirus 6 (one patient in 2018), Coxsackievirus A6 (one patient each in 2021 and 2022), Coxsackievirus A8 (one patient in 2019), Coxsackievirus A16 (one patient each in 2018 and 2022), and one patient each in 2018 for Coxsackievirus A2, A4, A9, and B3 and Echovirus 18.

## Discussion

The biannual peak pattern of AFM cases observed during 2014–2018 did not persist in 2020 or 2022. In 2020, nonpharmaceutical interventions for prevention of COVID-19 likely reduced the number of EV-D68 and other respiratory infections, which could have led to fewer cases of AFM (*5*–*8*). However, during the summer of 2022, sentinel surveillance among persons aged <18 years with acute respiratory illness detected increases in EV/RV and EV-D68 respiratory infections at levels not seen since 2018, suggesting that EV-D68 was widely circulating and causing respiratory illness in the United States during 2022 (*7*).

None of the patients with confirmed AFM since 2019 has received a positive EV-D68 test result, and only 39%–50% received a positive EV/RV test result. Diagnosing EV/RV infection among patients with AFM is challenging for several reasons. Respiratory specimens have the highest yield for detecting EV-D68, but because samples are typically collected at hospitalization several days to weeks after the start of a prodromal respiratory illness, the virus might no longer be present at the time of specimen collection (*1*–*3*). In addition, although most laboratories can test for EV/RV, further characterization (e.g., typing) is not available in most settings. CDC routinely performs EV/RV testing and, if results are positive, performs EV typing on specimens from patients with suspected AFM. Only 71% of confirmed cases during 2018–2022 had at least one specimen (respiratory, serum, cerebrospinal fluid, or stool) sent to CDC (CDC, unpublished data, 2018–2022); EV-D68 or other specific EVs might have been present in specimens that were not tested.

Historically, the clinical characteristics of confirmed AFM cases have varied among peak years (2016 and 2018) and nonpeak years (2015 and 2017), suggesting that AFM caused by EV-D68 might have a different clinical profile than AFM of other etiologies (*9*). Cases reported during 2019–2021 appeared similar to those reported during nonpeak years, with a lower proportion of antecedent respiratory illness or fever, upper limb involvement, and CSF pleocytosis, and a higher proportion of lower limb involvement, compared with cases in 2018. However, cases reported during 2022, when EV-D68 was circulating, did not follow this pattern: 2022 cases had a higher proportion of antecedent respiratory illness or fever, upper limb involvement, and CSF pleocytosis compared with cases during nonpeak years (2019 and 2021) and a lower proportion compared with cases during 2018.

Despite apparently increased EV-D68 circulation and EV-D68–associated respiratory disease among children, the reason why an increase in AFM cases did not occur in 2022 is unclear; possibly, EV-D68 viruses circulating in 2022 were less neurotropic or less likely to cause neurologic disease than were viruses circulating during 2014, 2016, and 2018. Another possibility is that infection with respiratory viruses including other RV/EVs, SARS-CoV-2, or respiratory syncytial virus that were frequently circulating in 2022 affected immune responses to EV-D68 and provided protection against neurologic disease (*6*). Data to support either of these hypotheses are lacking, and investigations are ongoing.

### Limitations

The findings in this report are subject to at least three limitations. First, this analysis was based on AFM cases reported to CDC and might underestimate the actual number of AFM cases in the United States. Second, clinical information is collected from a patient summary form typically completed by a health department and clinical records, which might contain incomplete data. Finally, 29% of cases did not have any specimens sent to CDC on which EV typing could be performed, limiting the ability to identify the specific EV associated with AFM.

### Implications for Public Health Practice

Current trends do not indicate when the next increase of AFM might be expected. Nonetheless, clinicians should be alert to the possibility of AFM among children with acute flaccid limb weakness and report to health departments when they suspect cases. In addition, to better understand causes for AFM, including the role of EVs and EV-D68, it is important that sufficient laboratory samples be collected to facilitate testing and typing of EVs.

## Footnotes

* EVs and RVs are closely related picornaviruses. Most available real-time reverse transcription–polymerase chain reaction tests for EV amplify a viral region that is highly conserved among EVs and RVs. Therefore, these tests do not distinguish among EVs and RVs, and additional testing, such as typing through sequencing, is needed to identify the specific virus that has been detected.

† All stool specimens submitted to CDC from persons with suspected AFM are tested for EV/RVs and poliovirus; any suspected AFM cases with specimens that test positive for poliovirus are considered polio cases and not AFM cases.

§ 45 C.F.R. part 46.102(l)(2), 21 C.F.R. part 56; 42 U.S.C. Sect. 241(d); 5 U.S.C. Sect. 552a; 44 U.S.C. Sect. 3501 et seq.
